# *Drosophila semaphorin2b* is Required for the Axon Guidance of a Subset of Embryonic Neurons

**DOI:** 10.1002/dvdy.23979

**Published:** 2013-04-20

**Authors:** Mark M Emerson, Jennifer B Long, David Van Vactor

**Affiliations:** Department of Cell Biology and Program in Neuroscience, Harvard Medical SchoolBoston, Massachusetts

**Keywords:** *Drosophila*, axon guidance, semaphorin

## Abstract

Background: The process of axon guidance is important in establishing functional neural circuits. The differential expression of cell-autonomous axon guidance factors is crucial for allowing axons of different neurons to take unique trajectories in response to spatially and temporally restricted cell non-autonomous axon guidance factors. A key motivation in the field is to provide adequate explanations for axon behavior with respect to the differential expression of these factors. Results: We report the characterization of a predicted secreted semaphorin family member, *semaphorin2b* (*Sema-2b*) in *Drosophila* embryonic axon guidance. Misexpression of *Sema-2b* in neurons causes highly penetrant axon guidance phenotypes in specific longitudinal and motoneuron pathways; however, expression of *Sema-2b* in muscles traversed by these motoneurons has no effect on axon guidance. In *Sema-2b* loss-of-function embryos, specific motoneuron and interneuron axon pathways display guidance defects. Specific visualization of the neurons that normally express *Sema-2b* reveals that this neuronal cohort is strongly affected by *Sema-2b* loss-of-function alleles. Conclusions: While secreted semaphorins have been implicated as cell non-autonomous chemorepellants in a variety of contexts, here we report previously undescribed *Sema-2b* loss-of-function and misexpression phenotypes that are consistent with a cell-autonomous role for *Sema-2b*. *Developmental Dynamics 242:861–873, 2013*. © 2013 Wiley Periodicals, Inc.

**Key Findings:**

- Misexpression of the secreted semaphorin *Sema-2b* in neurons results in specific axon guidance phenotypes.
- Both *Sema-2b* loss-of-function and misexpression phenotypes are congruent with a cell-autonomous role for *Sema-2b*.
- Novel axon guidance phenotypes caused by *Sema-2b* loss-of-function mutations are characterized.

## INTRODUCTION

A key component in the development of the nervous system is the process of axon guidance in which a neuron extends a process to its post-synaptic target. Several important families of axon guidance factors and receptors have been identified in a variety of systems and contexts to date, including the semaphorins/plexins, the ephrins/Eph receptors, the netrins/netrin receptors, and the slits/Roundabout receptors as prominent examples (Dickson, 2002). How these and other cues function together to specify the immensely complex wiring of the nervous system is of notable interest, especially the ability of an axon to make a unique decision as compared to its neighbor. This ability is thought to lie in both the differential expression of cell non-autonomous cues in the organism and cell-autonomous factors in the axon.

The evolution and creation of more complex nervous systems rely on the ability of the organism to enrich the informational landscape or generate novel molecular states for neurons in order to produce new guidance decisions and axonal trajectories. There are several examples where the differential expression of axon guidance factors immediately suggests how unique axon guidance behavior is achieved. For instance, in the *Drosophila* motoneuron system, several adhesion molecules display complementary expression in specific motoneurons and the muscles that they innervate (*connectin*, Fasciclin III, and *capricious*, for example) (Nose et al., 1992; Patel et al., 1987; Shishido et al., 1998; Snow et al., 1989). Likewise, in the *Drosophila* ventral nerve cord (VNC), expression of the Roundabout receptors correlates with the lateral position of longitudinal tracts relative to a highly localized source of the Slit repellant (Rajagopalan et al., 2000; Simpson et al., 2000). However, there are also examples where a selective axon guidance phenotype is found upon mutation of a broadly expressed axon guidance factor (Kolodziej et al., 1996; Krueger et al., 1996). This suggests that the differential expression of other factors must be responsible for this specific sensitivity.

Evolutionarily, it would be economical for organisms to add diversity to their neuronal connectivity, not only by evolving new guidance factors and associated pathways, but also by adding modulation to existing pathways. For example, several factors such as vertebrate *roundabout 3* and *Drosophila nedd4* and *commissureless* have been found to limit the amount of effective Slit ligand–Roundabout receptor signaling in a differential manner (either spatially or temporally) (Keleman et al., [Bibr b10],[Bibr b11]; Marillat et al., 2004; Myat et al., 2002; Sabatier et al., 2004; Tear et al., 1996). As vertebrates do not appear to have a *commissureless* gene and *Drosophilae* do not possess a *roundabout3* with a similar domain structure to the vertebrate gene, it suggests that they may have independently evolved new proteins to target the same signaling pathway. Recent studies suggest that there is another mechanism available to organisms to modulate specific axon guidance pathways without the genesis of novel proteins. Instead, it appears that ligands (including an example of semaphorins) that would normally be encountered in the environment by receptors on a growing axon can control the sensitivity of those same receptors when they are expressed in the same cell as the receptor (Carvalho et al., 2006; Moret et al., 2007). These ligands have already been selected to be binding partners for a particular receptor and so new protein–protein interactions need not be generated.

The *Drosophila* embryo provides a rich genetic and cellular context in which to identify axon guidance factors. Loss-of-function and enhancer trap screens have been successful at identifying axon guidance molecules in the *Drosophila* embryo. However, loss-of-function mutations in many of the differentially expressed genes fail to reveal a genetic requirement for these factors despite their suggestive expression and motifs. This has led to the hypothesis that there exists an unusually high level of over-specification present during the process of axon guidance in order to ensure developmental fidelity (Tessier-Lavigne and Goodman, 1996). Disruption in the differential expression of these genes by misexpression often produces interpretable phenotypes that are more penetrant than their cognate loss-of-function phenotypes (Nose et al., 1994). This property has been exploited by using the modular GAL4 system to conduct forward genetic screens based on misexpression as a means of recovering candidate genes with weak loss-of-function phenotypes in developmental processes outside of the nervous system (Rorth, 1996).

We performed a morphological screen to examine a subset of the axon pathways in the late *Drosophila* embryo (M. Emerson and D. Van Vactor, unpublished observations). Two of the mutants identified in this screen encode a member of the secreted semaphorin family, *Sema-2b*, which has been recently characterized (Wu et al., 2011). In agreement with this previous study, we characterize longitudinal axon guidance phenotypes with deviations in the medial-lateral axis. However, we identified additional loss-of-function *Sema-2b* phenotypes along the dorsal-ventral axis of the ventral nerve cord and in the motoneuron projections. These loss-of-function phenotypes and accompanying misexpression phenotypes are consistent with a cell-autonomous role for *Sema-2b*. Furthermore, the misexpression phenotypes of *Sema-2b* are similar to those observed in *plexinA* (*plexA*) and *semaphorin1a* (*sema1a*) loss-of-function mutants, suggesting that *Sema-2b* may normally antagonize Plexin/Semaphorin signaling. While secreted semaphorins have been implicated in a number of systems to act as cell non-autonomous repulsive axon guidance cues (Tran et al., 2007), both the misexpression and loss-of-function analysis of *Sema-2b* presented here suggests that *Sema-2b* may be able to exert cell-autonomous effects. Our findings complement previous observations of cell autonomy for secreted semaphorin during chicken embryogenesis (Moret et al., 2007), suggesting that this may be a common mechanism for regulating the specific activation of Plexin-Semaphorin signaling.

## RESULTS

### ME722 and EP2056 Misexpression Phenocopies Semaphorin/Plexin Loss-of Function Mutants

**Fig. 1:**
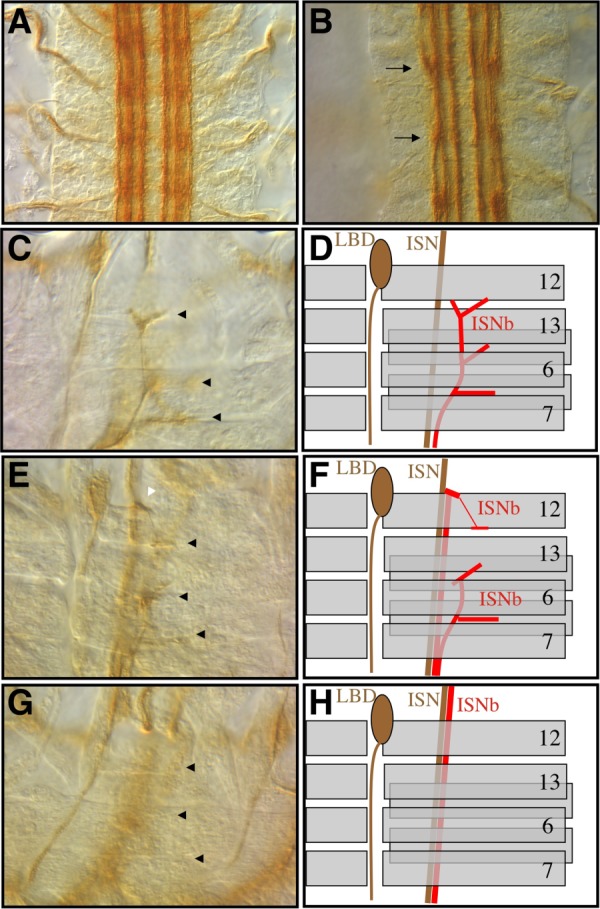
The misexpression phenotype of EP2056 and ME722 as seen in late stage-16 mAb 1D4-stained, dissected embryos. Embryos possess one copy of the Gal4 chromosome and one copy of EP2056 or ME722. **A,B:** Anterior is up and VNC longitudinal pathways are visualized. **C–H:** Anterior is left, dorsal is up, and the innervation of the ventral muscle domain by ISNb is visualized. A: A control embryo misexpressing an EP element with elav-Gal4, 24B-Gal4 that produces no phenotype. B: An EP2056 embryo upon misexpression with elav-Gal4, 24B-Gal4. Arrows point to deviation points of the lateral fascicle to the medial fascicle. C, E, G: Black arrowheads point to the three main innervation points of ISNb with the ventral muscles. C: The ME722 element with 24B-Gal4 showing the wild-type innervation for ISNb. E: The ME722 element with elav-Gal4 embryo showing an ISNb partial bypass phenotype. The white arrowhead points to the abnormal projection of a neuron off of ISN to innervate the cleft between muscle 12 and muscle 13. G: The ME722 element with elav-Gal4 embryo showing a full bypass ISNb phenotype with no innervation at any of the three normal ISNb innervation points. D, F, H: Schematic representations of the photomicrographs in C, E, and G showing the mAb 1D4-positive LBD neuron as well as the ISN and ISNb pathways. ISNb is shown in red and prominent ventral muscles are numbered.

Several groups have used a misexpression strategy in a forward genetic screen context to identify axon guidance molecules in the *Drosophila* embryo and larvae (Kraut et al., 2001; McGovern et al., 2003; Mindorff et al., 2007; Umemiya et al., 2002; Zlatic et al., 2009). The modular misexpression system makes use of a collection of randomly inserted P-elements that contain several UAS binding sites, allowing for specific temporal and spatial misexpression of potential downstream genes when crossed to a Gal4 driver line. Using a Gal4 driver line that expresses in both post-mitotic neurons and embryonic muscles (an elav-Gal4, 24B-Gal4 recombinant line), two lines (ME722 and EP2056) with identical phenotypes were identified in an immunohistochemical screen using an antibody (mAb 1D4) that labels all motoneurons and a subset of longitudinal pathways in the VNC (M. Emerson and D. Van Vactor, unpublished observations). Out of 600 lines screened, 16 lines in total were found to have phenotypes; only ME722 and EP2056 possessed the constellation of phenotypes described below. Control late stage-16 embryos have five mAb 1D4-positive longitudinal axon pathways bilaterally represented in the VNC, three of which are found in the same dorsal-ventral plane (Fig. 1A). Misexpression of ME722 or EP2056 often caused the outermost fascicle of the three to move medially to join the middle longitudinal fascicle (Fig. 1B, Table 1). Axons of ME722 and EP2056 misexpression embryos continued to extend along the anterior-posterior axis rejoining the third fascicle in the next segment of the embryo. This phenotype is very similar to that reported for loss-of-function mutations that disrupt Plexin/Semaphorin signaling, such as *plexinA* and *sema1a* (Winberg et al., 1998b; Yu et al., 1998).

**TABLE 1:** Quantitation of Misexpression Phenotypes

Genotype	N^a^	WT ISNb	ISNb bypass	Third fascicle
ME722/+;elav/+	62	31 (50%)	31 (50%)	ND
ME722/+;24B/+	216	216 (100%)	0	ND
ME12/+;Gr12/+	140	140 (100%)	0	ND
ME475/+;Gr12/+	126	126 (100%)	0	ND
EP2056/1407	146	15 (10.3%)	131 (89.7%)	93.3% (15)
UAS-Sema2a/1407	169	131 (77.5%)	38 (22.5%)	0% (16)
1407/+; UAS-Sema2b(FL.1)/+;	188	75 (39.9%)	113 (60.1%)	92.3% (26)
1407/+; UAS-Sema2b(FL.2)	224	174 (77.7%)	50 (22.3%)	4.3% (23)
1407/+; UAS-PlexA/+	185	182 (98.4%)	3 (1.6%)	0%
1407/+; UAS-PlexB/+	183	180 (98.4%)	3 (1.6%)	0%
1407/UAS-Sema1a	184	163 (88.6%)	21 (11.4%)	0%

a N refers to the total number of segments scored for a particular genotype. Numbers in each column are the actual number of segments that scored for a particular phenotype and the percentage of the total number of segments scored is shown in parentheses.

Defects were also observed in the ISNb motoneuron pathway of ME722 and EP2056 misexpression embryos. In control embryos, mAb 1D4-positive ISNb motoneurons are fasciculated with the ISN pathway as they exit the VNC. Upon reaching the ventral muscle field, wild-type ISNb motoneurons defasciculate from ISN and enter the ventral muscle field, forming a number of stereotyped synaptic connections (Fig. 1C, D). In contrast, in ME722 and EP2056 misexpression embryos the ISNb often remains fasciculated with the ISN axons and bypasses the ventral muscle domain. ISNb was sometimes observed innervating the ventral muscles from an abnormal defasciculation point off of ISN, near muscle 12 (Fig. 1E, F). Occasionally, a full ISNb bypass phenotype was observed with none of the ISNb axons entering the ventral field, rather, making contacts with more dorsal muscle targets such as muscle 4 (Fig. 1G, H). No defects were seen in midline crossing or in ISN motoneuron guidance, suggesting a substantial degree of specificity in the misexpression phenotypes. Furthermore, muscle morphology appeared normal, suggesting that the phenotypes were not secondary to a muscle defect. On the basis of the specificity of these phenotypes, we decided to investigate the molecular nature of these alleles.

### ME722 and EP2056 Direct the Misexpression of Sema-2b

**Fig. 2:**
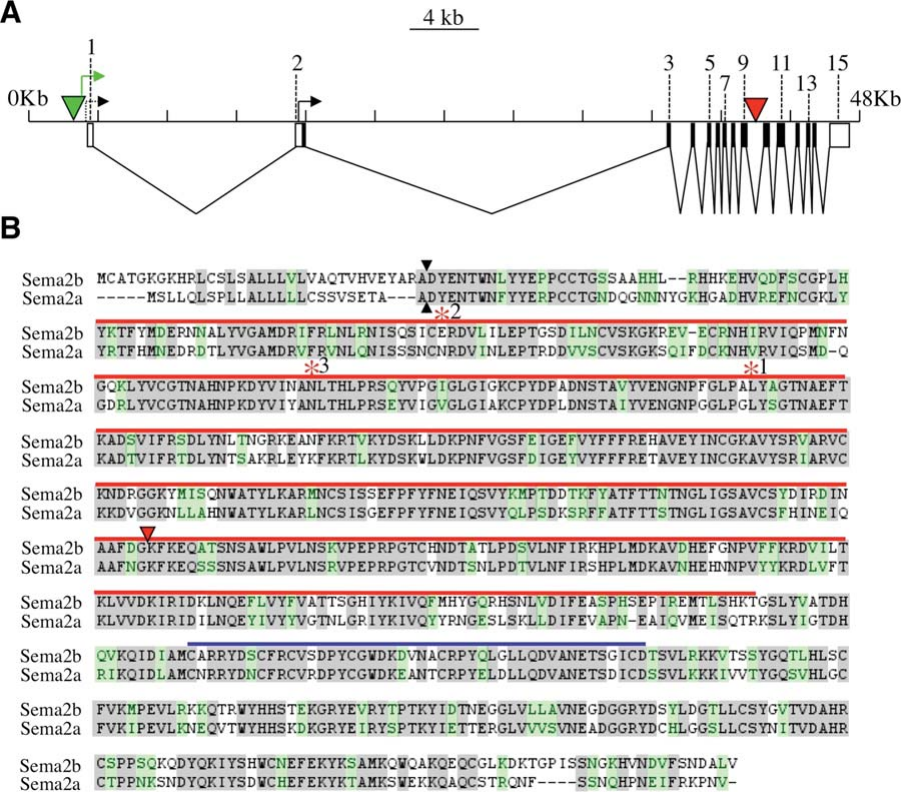
Genomic and transcript analysis of the *semaphorin2b* gene **A**: A schematic of the *semaphorin2b* locus. The horizontal line represents the genomic region located at 53C1, with the small vertical lines marking 4-kilobase intervals. Rectangles located beneath the line represent exons defined by cDNA analysis with untranslated regions shown with open rectangles and coding regions shown with black rectangles. The two black arrows represent points of transcription initiation as defined by cDNA analysis. The green triangle represents the insertion point of EP2056 and ME722 and the green arrow shows the direction of misexpression transcription initiated by Gal4. The red triangle represents the insertion point of the f02042 piggyBac element. **B:** A lineup of the predicted Sema2a and Sema2b proteins. Potential signal sequence cleavage sites are shown with black arrowheads. Identical amino acids are shaded grey and similar amino acids are shaded green. The position of the semaphorin domain is marked with a red line and the position of the PSI domain is shown with a blue line. The location of the insertion point of f02042 in the genome relative to the primary amino acid sequence of *sema2b* is shown with a red triangle. RT-PCR analysis suggested the presence of several splice isoforms with altered amino acid sequence of the Sema2b protein. The effects of three such isoforms that were used to generate UAS constructs are shown here. The asterisk denotes the last amino acid in the predicted protein that matches the wild-type sequence before it diverges. Truncation 1 was caused by part of the intron after exon 8 being included in the cDNA and a novel 10 amino acids being added to the partial Sema2b polypeptide. Truncation 2 resulted from exon 4 splicing directly to exon 10 and skipping exons 5–9. Sixteen novel amino acids were added to the partial Sema2b polypeptide. Truncation 3 resulted from exon 6 splicing to the last 8 nucleotides of exon13 and skipping exons 7–12. This resulted in a partial Sema2b polypeptide with 19 additional amino acids.

In order to identify the gene misexpressed by ME722 and EP2056, we used inverse PCR to identify the sequence adjacent to the inserted P-element. The recovered sequence positioned ME722 just upstream of the transcriptional start site of the *Sema-2b* gene, a recently characterized secreted semaphorin family member (Fig. 2A) (Sweeney et al., 2011; Wu et al., 2011). Inverse PCR performed by the Berkeley *Drosophila* Genome Project on the EP2056 line identified its insertion site to be located within a few nucleotides to the ME722 insertion site. The orientation of each P-element was determined to be such that the nearest predicted gene to be misexpressed upon Gal4 transactivation is *Sema-2b*.

To determine if Gal4 activated transcription from the ME722 P-element was capable of misexpression of the *Sema-2b* transcript, we performed reverse-transcriptase polymerase chain reaction (RT-PCR) on cDNA from embryos with the ME722 and elav-Gal4 P-elements. A hybrid cDNA composed of sequences from the ME722 P-element and *Sema-2b* was amplified and confirmed by sequencing. Sequence alignment of full-length Sema-2b protein showed it to be highly homologous with the Sema-2a protein (Fig. 2B). Both proteins contain a predicted signal sequence, a full-length semaphorin domain, and a plexin-semaphorin-integrin (PSI) domain.

### ISNb Axon Guidance Defects Are Caused by Misexpression of *Sema-2b* in Neurons and Not in Muscles

The most closely related semaphorin molecule to Sema-2b is Sema-2a. *Sema-2a* is expressed within the VNC and in thoracic muscle 33 (Kolodkin et al., 1993). Misexpression of *Sema-2a* on muscles can prevent innervation of those muscles by their normal axons, suggesting that it can act as a repellant (Matthes et al., 1995). Likewise, loss-of-function mutations in *Sema-2a* lead to ectopic innervations of motoneurons onto muscle, again consistent with a role as a secreted repellant acting in a cell non-autonomous fashion during motoneuron axon guidance and targeting. However, whether the defect in axon guidance was due to loss of *Sema-2a* expression in muscles or neurons was not assessed (Winberg et al., 1998a).

Given the similarity between the Sema-2a and Sema-2b proteins, it seemed likely that the misexpression phenotypes observed with *Sema-2b* could be best interpreted in light of *Sema-2a*'s non-cell-autonomous and repellent activity. Thus, we initially hypothesized that the ISNb motoneuron phenotype we observed was due to *Sema-2b* misexpression in muscles repelling ISNb axons in a non-cell-autonomous manner. However, when we tested the model by misexpressing *Sema-2b* solely in muscles using 24B-Gal4, no ISNb phenotype was observed (Table 1). Surprisingly, misexpression of *Sema-2b* in neurons wholly recapitulated the ISNb phenotype (Table 1). The ability of neuronal *Sema-2b* to redirect motoneurons along abnormal trajectories suggested that *Sema-2b* can act in a cell-autonomous manner (affecting only the axons that express it) or very locally between axons in a manner that is not recapitulated by extracellular expression from underlying muscles. This is consistent with a previous study of *Sema-2b*, which also revealed autonomous properties of *Sema-2b* in the ventral nerve cord (Wu et al., 2011). This is a very different function than has been previously described for other secreted semaphorins, most notably the closely related *Sema-2a*.

### Neuronal Expression of Full-Length *Sema-2b* Recapitulates the Misexpression Phenotypes of ME722 and EP2056

To prove that the observed misexpression phenotypes of the EP2056 and ME722 P-elements were indeed caused by *Sema-2b*, P-element constructs were made with *Sema-2b* cDNAs driven by UAS elements. Using RT-PCR, a major transcript encoding full-length *Sema-2b* was identified as well as several *Sema-2b* transcripts that encoded truncated products. As it seemed possible that misexpression of a normal truncation product may be also able to function as a cell-autonomous inhibitor, transgenic constructs encoding both the full-length and three truncated forms of the Sema-2b protein were placed under the control of UAS enhancer sequences. To assess whether full-length or truncated Sema-2b is responsible for the misexpression phenotypes we identified in our primary screen, the UAS lines were crossed to the neuronal driver 1407-Gal4 in an otherwise wild-type background. Two independent insertions (FL.1 and FL.2) of the full-length *Sema-2b* cDNA were tested and both had significant levels of ISNb bypass, though FL.1 had a three-fold higher penetrance than FL.2 (60.1 to 22.5%, Table 1) and was closer to the level of bypass seen with the original ME722 P-element insertion (89.7%). Embryos from both lines also had a third fascicle phenotype identical to that observed in the original P-element lines (ME722 and EP2056), though again, one of the lines had a much higher penetrance than the other (92.3 to 4.3%, Table1). The original P-element insertion, ME722, had a third fascicle phenotype penetrance closer to that observed with FL.1 (93.3%). Effects of genomic position on expression level are likely to account for the difference in phenotype penetrance we observed for the two transgenic lines, as is typical for random transposon insertion sites. In order to determine if this was indeed the case, the expression level of *Sema-2b* RNA induced in embryos of each line by elav-Gal4 was quantified by qRT-PCR. Relative to wild-type embryos, those with the FL.1 transgene possessed 1.67 ± 0.185 fold more *Sema-2b* RNA (*P* < 0.01) while those with the FL.2 transgene had 1.187 ± 0.183 fold more *Sema-2b* RNA (not significant, *P* = 0.13). Thus, the phenotypic penetrance differences observed between these two full-length *Sema-2b* lines correlates with the induced expression level of *Sema-2b* RNA. Lastly, three *Sema-2b* cDNAs identified by RT-PCR and predicted to encode truncated Sema-2b products, had no effect when neuronally misexpressed (data not shown, Fig. 2; see Experimental Procedures section for further details on these isoforms). These experiments suggest that the misexpression phenotype is not the result of expression of a dominant-negative Sema-2b protein, but from a full-length Sema-2b isoform.

### Neuronal Misexpression of Other Semaphorins and the Plexins

Given the close sequence homology between the Sema-2a and Sema-2b proteins, it seemed likely that these two proteins might have similar biochemical activities. In order to test if *Sema-2a* could have cell-autonomous properties, we misexpressed *Sema-2a* in neurons. Like *Sema-2b*, *Sema-2a* misexpression in neurons also had a significant ISNb bypass phenotype, though unlike *Sema-2b*, no third fascicle phenotypes were observed (Table 1). The similarity of the *Sema-2a* and *Sema-2b* ISNb misexpression phenotypes stands in contrast to other axon-targeting experiments that have shown disparate activities in the CNS for these two genes (Wu et al. 2011). This suggests that the cellular context may dictate the effects of these molecules. In addition, other semaphorin/plexin molecules were tested. As previously shown, misexpression of a transmembrane semaphorin, *sema1a*, also results in an ISNb bypass phenotype, though it is not as robust as that of *Sema-2b* or *Sema-2a* misexpression (Table 1) (Yu et al., 1998). In contrast, misexpression of either of the two reported semaphorin receptors in *Drosophila, plexA* and *plexinB* (*plexB)*, did not cause an ISNb bypass phenotype or a third fascicle phenotype (Table 1). In conclusion, neuronal misexpression of all of the secreted and transmembrane semaphorins tested here resulted in an ISNb bypass phenotype, while expression of the Plexin receptors did not.

### Sema-2b mRNA Expression

**Fig. 3:**
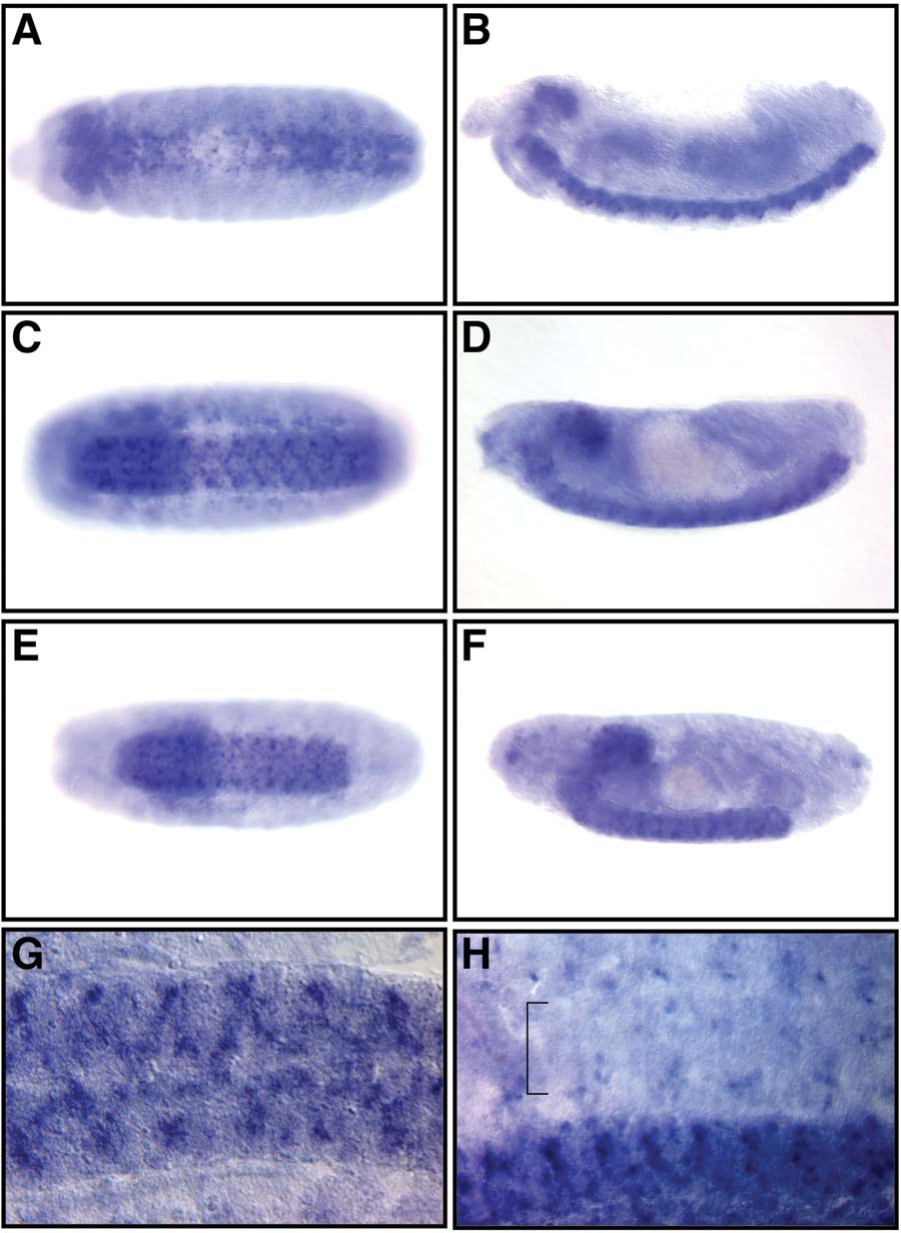
*Sema-2b* mRNA localization. A–F: Whole-mount photomicrographs of embryos processed for RNA *in situ* hybridization using *Sema-2b* probes. Anterior is to the left. A,C,E: Ventral views of an embryo centered on the VNC. B,D,F: Lateral views of the same embryos shown in the ventral view. **A,B:** Stage 13. **C,D:** Stage 15. **E,F:** Late stage 16. **G:** A dissected late stage-16 embryo focused on the VNC. **H:** A dissected late stage-16 embryo with both the VNC and the periphery represented. The bracket marks the ventral muscle domain.

To determine the expression pattern of *Sema-2b*, we generated antisense RNA in situ hybridization probes to *Sema-2b*. *Sema-2b* mRNA was detected primarily in the VNC in a restricted and segmentally repeated pattern beginning early in neurogenesis and continuing till at least the end of embryogenesis (Fig. 3A–G). No signal was detected in muscles, but expression was detected in a small number of peripheral cells whose positions were consistent with being sensory neurons (Fig. 3H). Elav-Gal4/ME722 embryos had elevated *Sema-2b* mRNA expression in post-mitotic neurons compared to wild-type embryos, providing additional evidence that *Sema-2b* is the gene misexpressed by ME722 (data not shown).

### Loss-of-Function Alleles of *Sema-2b* Cause Specific Motoneuron Defects

**Fig. 4:**
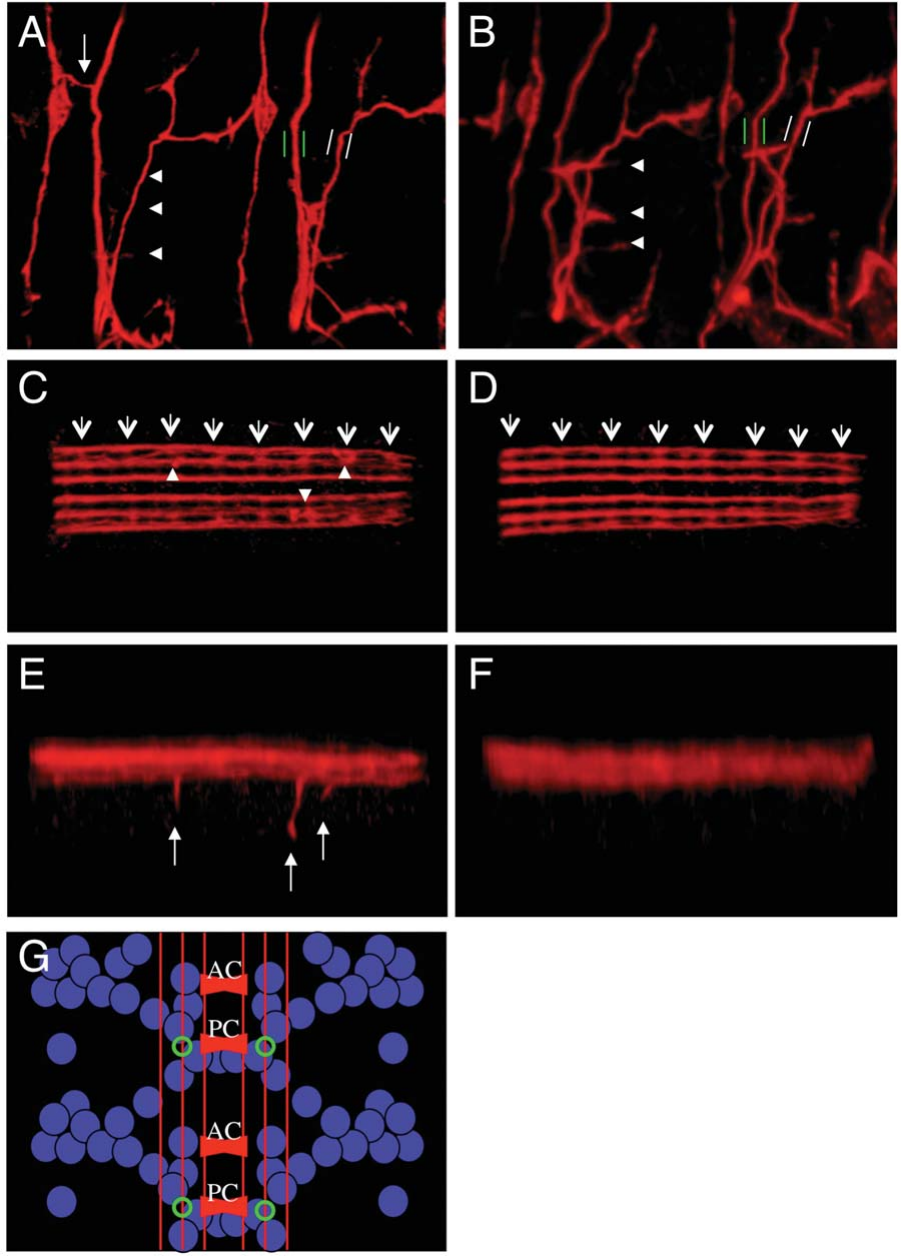
*Sema-2b* loss-of-function phenotypes. A–F: Confocal microscopy of late stage-16 embryos stained with mAb 1D4 antibody in red. A,C,F: f02042 homozygous embryos. B, D, E: f02042[Ex9] homozygous embryos. **A,B:** Views of two segments showing the ventral muscle domain and its innervation by ISNb. White arrowheads mark the normal innervation points by ISNb of the ventral muscles of one of the segments. Green parallel bars mark the ISN motoneurons and white parallel bars mark the SNa motoneurons to show their relative thicknesses in the two genotypes. White arrow shows an ectopic innervation from ISN to the LBD neuron. **C,D:** Ventral view of the VNC. White arrows mark the position of the posterior commissure of each segment and white arrowheads mark the position that ectopic ventral projections leave the medial fascicle. **E,F:** Lateral view of the same embryos as in C and D with arrows pointing to the ectopic ventral projections. **G:** A schematic of the mRNA expression of *sema2b* (blue), the mAb 1D4 fascicles (red), and the position where ectopic ventral projections are observed (green).

To examine the endogenous function of *Sema-2b*, we acquired a line with a piggybac insertion (f02042) in intron 9 of the *Sema-2b* gene, which is located in the portion of the gene encoding the C-terminal portion of the semaphorin domain (Fig. 2A). To determine if *Sema-2b* functions in motoneuron axon guidance, we examined late-stage embryos homozygous for the f02042 insertion using mAb 1D4 immunohistochemistry. We found defects in ISNb axon guidance within close proximity to their ventral muscle targets. Approximately, 10% of segments had ISNb defects, and within these segments only some of the ISNb neurons showed defects in axon guidance (Fig. 4A, Table 2). No defects were observed in other motoneuron branches. This suggests that *Sema-2b* is necessary for proper ISNb axon guidance.

**TABLE 2:** Quantitation of Sema-2b Loss-of-Function Phenotypes

Genotype	Genetic description	ISNb bypass(n^a^)	E.V.P^c^/Embryo(N^b^)
F02042/f02042	*sema2b* transposon mutant	9.2% (1313)	0.83 (157)
Df7142/Df7142	*sema2b* deficiency mutant	8.6% (421)	0.87 (39)
D05894/d05894	*sema2b* isogenic wild-type	0.9% (423)	ND
W1118	Wild-type	1.5%(334)	ND
Ex9/f02042	Precise Excision Het.	0.7% (430)	0.05 (38)
ExMN3/f02042	Precise Excision Het.	2.3% (397)	0.08 (36)
Ex9/Ex9	Precise Excision Hom.	1.6% (312)	0.14 (28)
ExMN3/ExMN3	Precise Excision Hom.	0.4% (446)	0.03 (39)

a n is the total number of segments scored. N is the number of embryos scored.

E.V.P. refers to Ectopic Ventral Projections, which were scored as 1D4-positive. Axons were detected ventral to their normal location. ISNb bypass phenotypes were scored as any lack of innervation from the ISNb nerve that was accompanied by a concomitant increase in ectopic projections from the ISN. Het., heterozygotes; Hom., homozygotes.

The f02042 element could be acting as a loss-of-function allele of *Sema-2b* or it could be creating a neomorphic allele of *Sema-2b* by creating a premature truncation. To distinguish between these two possibilities, we obtained a deficiency (Df7142) that was generated by recombining f02042 with another piggybac element located 5 prime to *Sema-2b*, deleting the intervening sequence. This allele is expected to lack the transcription initiation site, the starting methionine and all of the coding sequence up to the f02042 insertion site, presumably creating a *Sema-2b* null allele (Parks et al., 2004). While some of the reported deficiency alleles created by this method have later been shown to not actually create a deficiency, at least two lines of evidence support Df7142 as an actual deficiency allele (Cook 2012). First, it has previously been tested in a complementation assay with an allele of *wcd*, a gene expected to be deleted in Df7142, and failed to complement this mutation (Fichelson et al., 2009). Secondly, the abundance of *Sema-2b* RNA in embryos of a Df7142/CyO line was tested by qRT-PCR and found to be significantly less than that of wild-type embryos (*P* < 0.05). We examined embryos homozygous for Df7142, and found they had similar ISNb defects, both qualitatively and quantitatively, to f02042 mutant embryos, suggesting that f02042 is likely to be a null allele of *Sema-2b* (Table 2).

Df7142 was created from f02042, leaving open the possibility that a mutation other than the one in *Sema-2b* could be causing the ISNb defects. To control for strain background, another insertion line (d05894) located 3 prime to the *Sema-2b* gene and generated in an isogenic background to f02042 was examined but had negligible ISNb defects (Table 2). To definitively confirm that the defect in *Sema-2b* was indeed the cause of the ISNb defects, we generated precise excision alleles of the f02042 piggybac element. Quantification of the ISNb defects in two independent excision alleles showed a level of ISNb defects comparable to that seen in wild-type embryos, suggesting that the piggybac insertion was indeed the causal mutation of the ISNb phenotype (Table 2).

### Loss-of-Function Alleles of *Sema-2b* Cause Specific Longitudinal Axon Guidance Defects

Examination of the longitudinal pathways labeled by mAb 1D4 in the VNC of *Sema-2b* mutants revealed specific defects in the medial fascicle of these longitudinal pathways. Normally, these axons project anteriorly without deviating significantly in the medial-lateral or dorsal-ventral direction. In f02042 and Df7142 homozygous mutants, these tracts appeared more disordered in the medial-lateral plane than precise excision alleles, with axons found between the middle and the lateral longitudinal tracts (Fig. 4C, D). These defects are consistent with those reported for the independently derived and previously reported allele of *Sema-2b* (Wu et al., 2011).

More striking than the medial-lateral deviations in axon guidance were those in the dorsal-ventral axis of the embryo. Large axon bundles were found to leave the middle longitudinal tract and project ventrally and occasionally also laterally within the VNC (Fig. 4C, E). These projections occur from the ventral side of the tract and appear to have no bias as to where they occur along the general anterior-posterior axis of the embryo. However, these projections always occur at the level of the posterior commissure within the segment, suggesting that this may be a choice point for longitudinal axon guidance. We refer to this previously undescribed phenotype as an ectopic ventral projection. To confirm that these defects were caused by *Sema-2b* loss-of-function, the precise excision alleles of f02042 were examined and found to have comparable levels of ectopic ventral projections to wild-type flies (Fig. 4D, F; Table 2).

### Sema-2b-Positive Neurons Are Affected by Sema-2b Loss-of-Function Mutations

To assess whether *Sema-2b*-positive axon pathways are affected by *Sema-2b* loss-of-function alleles, a previously characterized transgenic line carrying *Sema-2b* upstream regions driving the expression of the human Tau protein as well as the Myc epitope (Sema-2b-Tau-myc) was introduced into both the f02042 and f02042 precise excision lines. This line drives strong expression in a small subset of *Sema-2b*-positive neurons with mainly longitudinally projecting axons that also cross the midline in the anterior commissure as well as some neurons in the brain (K.A. Senti and B.J. Dickson unpublished results, (Rajagopalan et al., 2000; Wu et al., 2011). If these Sema-2b-positive neurons were affected by *Sema-2b* loss-of-function, this would be further evidence that *Sema-2b* has a cell-autonomous function.

**Fig. 5:**
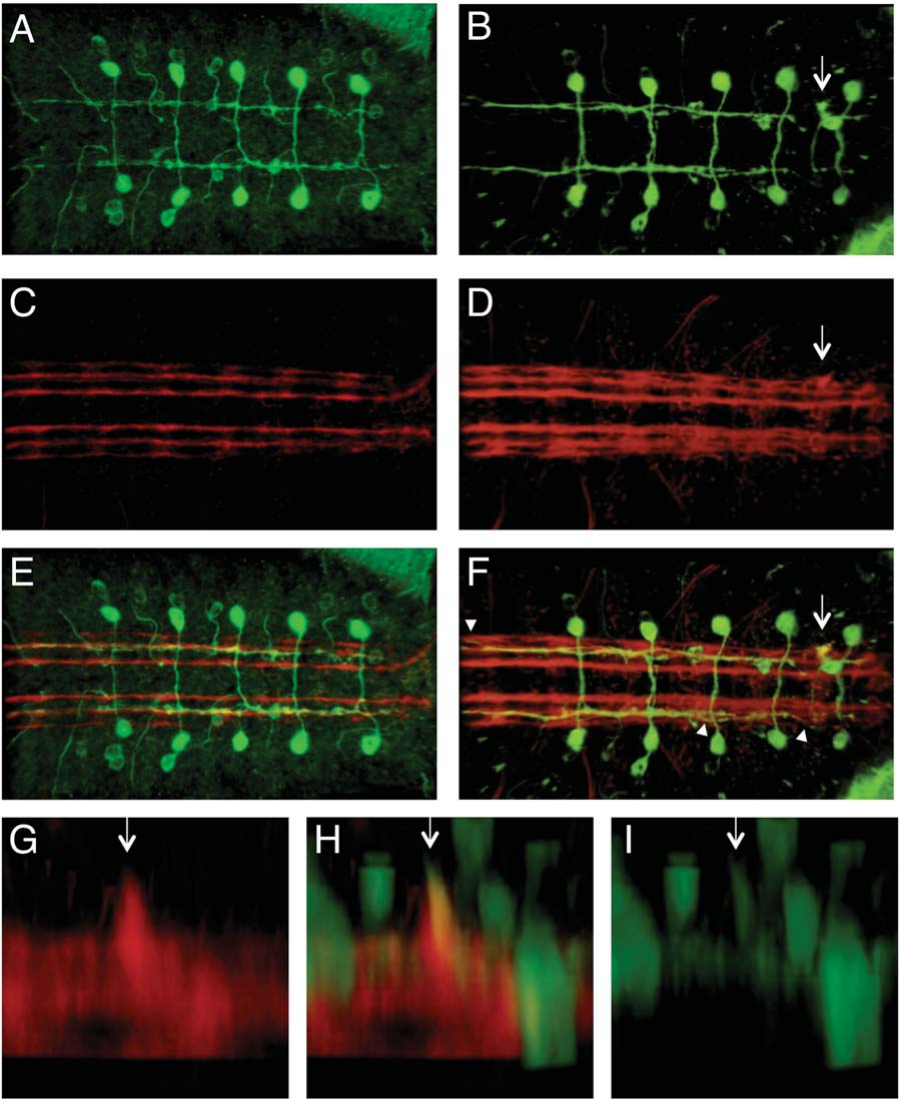
Visualization of *Sema-2b*-positive neurons in a *Sema-2b* loss-of-function background. Confocal microscopy of late stage-16 embryos expressing a sema2b-tau-myc transgene stained with anti-tau (green) and mAb 1D4 (red). Arrows point to an ectopic ventral projection. Arrowheads point to positions where the medial fascicle joins the lateral fascicle. Anterior is to the left. A, C, E: f02042[Ex9] homozygous embryos. B, D, F–I: f02042 homozygous embryo. **A, B:** Anti-tau. **C,D:** mAb 1D4. **E,F:** Merged z projection of the red and green channels. **G–I:** A lateral view of the VNC of a f02042 homozygous mutant embryo with an ectopic ventral projection.

We first examined the axons labeled in the Sema-2b-Tau-myc enhancer line in one of the precise excisions of f02042 using confocal microscopy. As reported previously, *Sema-2b*-positive axons were found to extend longitudinally as part of the mAb 1D4-positive middle fascicle, deviating neither to the more medial or lateral fascicle throughout the anterior-positive extent of the VNC (Fig. 5A). In stark contrast to precise excision embryos, there were two obvious phenotypes that were observed in the f02042 homozygous embryos. The first was a deviation of the *Sema-2b* axons from the second fascicle to the more lateral third fascicle, a lateral detour defect previously identified by Wu et al. (Fig. 5B). This phenotype likely contributes to the ectopic mAb 1D4-positive axons that were observed between the medial and lateral fascicles in *Sema-2b* mutant embryos (Figs. 4C, 5D). Interestingly, this *Sema-2b* loss-of-function phenotype is complementary to the misexpression phenotype where axons from the third fascicle move to join the second fascicle (Fig. 1B). This suggests that *Sema-2b* is both necessary for keeping axons in the second fascicle and sufficient to recruit axons from the third fascicle to join the second fascicle. The second *Sema-2b* phenotype observed with the Sema-2b-Tau-myc was ectopic ventral projections originating from the medial fascicle at the level of the posterior commissure, just as was seen with mAb 1D4 (Fig. 5B, D). Indeed, all of the ectopic ventral projections can be co-labeled to some extent with both mAb 1D4 and the Sema-2b-Tau-myc reporter. A lateral view of the VNC reveals that half of the mAb 1D4-positive projection also expresses the Sema-2b transgene (Fig. 5G–I). It appears that the *Sema-2b*-positive axon(s) are located at the more distal end of the projection, suggesting that a defect in the *Sema-2b*-positive axon could be the primary cause of the phenotype.

## DISCUSSION

The ability of adjacent neurons to make unique axon guidance decisions relative to each other is dependent on the differential expression and activity of axon guidance factors and cell adhesion molecules between these neurons as well as spatially localized, non-cell-autonomous ligands (Lowery and Van Vactor, 2009). While the immense complexity of some nervous systems suggested that many signaling pathways are employed during development, it has become increasingly clear that several conserved pathways are used repeatedly to shape the development of different neural circuits throughout the embryo. Specific mechanisms to modulate these pathways allows for a greater complexity of available signaling states and therefore for correspondingly more complex circuits to form.

We adopted a misexpression approach in the *Drosophila* embryo to aid in the recovery of differentially expressed factors that contribute to the specificity of individual axon guidance decisions. One of these factors is predicted to encode a member of the secreted semaphorin family, *Sema-2b*. In *Drosophila*, there are six identified semaphorins encoded in the genome: four transmembrane semaphorins (*sema1a, 1b, 3a*, and *5c*) and two secreted semaphorins (*Sema-2a* and *2b*) (Khare et al., 2000; Winberg et al., 1998b). Plexins are structurally related to semaphorins and act as transmembrane receptors for the semaphorins (Tamagnone et al., 1999; Winberg et al., 1998b). Of the two *Drosophila* plexins, *plexA* has been shown to transduce transmembrane semaphorin signals while *plexB* transduces secreted semaphorin signals (Ayoob et al., 2006; Winberg et al., 1998b; Wu et al., 2011). A number of downstream signaling components have also been identified in *Drosophila* including *off-track*, Mical, Rac, *nervy*, and Gyc76C (Ayoob et al., 2004; Hu et al., 2001; Terman and Kolodkin, 2004; Terman et al., 2002; Winberg et al., 2001). However, there are still open questions about how semaphorins, plexins, and their downstream components function together to drive specific neuronal connections.

In the *Drosophila* embryo, the roles of several semaphorins and plexins have been investigated. ISNb axon guidance is qualitatively affected in the same way in *sema1a, plexA*, and *plexB* loss-of-function mutants. In all three mutants, ISNb fails to appropriately innervate its normal ventral muscle targets and instead continues to project dorsally with the ISN (an ISNb bypass phenotype) (Ayoob et al., 2006; Winberg et al., 1998b; Yu et al., 1998). Similarly, the individual misexpression of *sema1a, plexA*, and *plexB* also lead to ISNb bypass phenotypes or ISNb stall phenotypes that can be interpreted as bypass phenotypes (Winberg et al., 1998b; Yu et al., 1998; and this study). Given that *sema1a, plexA*, and *plexB* appear to be expressed by most neurons, it is unclear why loss-of-function mutations in these genes cause specific phenotypes, and also if the semaphorin or the plexins are acting as the receptor in the ISNb neurons. Similarly, in the VNC, loss-of-function mutations in both *sema1a* and *plexA* lead to the same phenotype with the outermost mAb 1D4 fascicle occasionally moving medially to join the middle fascicle. Here again, the fact that only a subset of longitudinal axons are affected, despite the pan-neuronal expression of these two components, suggests that there is likely to be other genetic components functioning to provide specificity.

Most studies to date have interpreted semaphorins as non-cell-autonomous factors in the context of axon guidance. *In vitro*, a clear non-cell-autonomous function of semaphorins was revealed in their initial characterization, as exogenous protein applied to growing axons induces a repellant response (growth cone collapse) (Luo et al., 1993). *In vivo*, the analysis of semaphorin loss-of-function phenotypes is harder to interpret in terms of autonomy given that multiple anatomical loci often express a given semaphorin. However, several studies have directly implicated the transmembrane semaphorin, *sema1a*, as having a cell-autonomous function (Cafferty et al., 2006; Godenschwege et al., 2002; Komiyama et al., 2007). A cell-autonomous role for secreted semaphorins is intuitively less obvious given the lack of a cell association motif such as a transmembrane domain or a GPI-anchor.

For vertebrate secreted semaphorins, only one study has strongly suggested a cell-autonomous function (Moret et al., 2007). In this study, electroporation of motoneurons of the chick spinal cord allowed for misexpression and loss-of-function studies of the secreted semaphorin, Sema3A, and its effects on motoneuron axon guidance. A cell-autonomous function for Sema3A was deduced from these experiments and the molecular mechanism for its activity was shown to be regulation of the cell surface expression of the vertebrate semaphorin receptor, Neuropilin. In this way, the expression of Sema3A by a neuron could regulate the sensitivity of that neuron to non-cell-autonomous presentation of Sema3A and perhaps other semaphorins as well (Moret et al., 2007). An analogous cell-autonomous function has also been proposed for an ephrin ligand in regulating an Eph receptor, suggesting that this may be a more general mechanism used to regulate the growth cone's sensitivity to axon guidance factors (Carvalho et al., 2006).

In *Drosophila*, two secreted semaphorins, *Sema-2a* and *Sema-2b*, have been genetically investigated. *Sema-2a* is expressed in a single muscle in the periphery as well as a subset of neurons in the VNC. Because of its expression in a muscle and its secreted protein structure, it was hypothesized to be a non-cell-autonomous factor for motoneurons (Kolodkin et al., 1993). Indeed, the misexpression of *Sema-2a* in muscles was capable of preventing normal innervation of muscles by their cognate motoneurons suggesting it can act as a non-cell-autonomous chemorepellant (Matthes et al., 1995). However, recent analysis of axon guidance within the CNS suggests that the highly related *Sema-2b* may display highly context-dependent autonomy behavior (Sweeney et al., 2011; Wu et al., 2011). Our findings strongly support a cell-autonomous function in three ways. First, misexpression of *Sema-2b* in neurons generates robust motoneuron phenotypes in the vicinity of muscles, but strong misexpression of *Sema-2b* in muscles has no effect on motoneuron axon guidance. Second, *Sema-2b*-positive neurons in the VNC themselves are strongly affected by *Sema-2b* loss-of-function mutations. Third, misexpression of *Sema-2b* phenocopies *plexA* and *sema1a* loss-of-function phenotypes. Therefore, our data are consistent with a model where in certain contexts *Sema-2b* does not promote but rather down-regulates Plexin-Semaphorin signaling, analogous to the cell-autonomous function proposed for Sema3A in vertebrate motoneuron axon guidance **(**Moret et al., 2007**)**. This model would propose that in *Sema-2b* loss-of-function mutants, PlexA/Sema1a signaling is hyperactivated in a subset of neurons and would predict that manipulations that hyperactivate this signaling in a wild-type context might phenocopy the *Sema-2b* loss-of-function phenotype. Our preliminary results suggest that neuronal misexpression of PlexA or Sema1a does phenocopy the *Sema-2b* ISNb loss-of-function phenotype, though, for both Sema-2b and plexA/Sema1a misexpression and loss-of-function alleles, an ISNb bypass phenotype is observed, so this may not be a valid phenocopy (data not shown). However, misexpression of PlexA or Sema1a does not phenocopy the ectopic ventral projection phenotype of *Sema-2b* mutants (data not shown). However, it is unclear if misexpression of these proteins is really sufficient to overcome the inhibition by *Sema-2b* and/or whether misexpression is really equivalent to hyperactivation. Further genetic and molecular studies will be necessary to test this hypothesis.

**Fig. 6:**
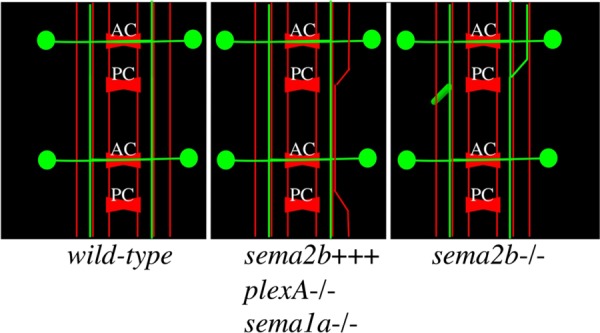
Schematic of *Sema-2b* phenotypes. A schematic of a stage-16 VNC with the three most prominent mAb 1D4 fascicles (red) and some of the Sema2b-Tau-Myc neurons (green) depicted. The wild-type VNC has Sema2b-Tau-Myc axons traversing in the middle mAb 1D4 fascicle after crossing through the anterior commissure. The middle schematic shows the effects of neuronal misexpression of *Sema-2b* and loss-of-function mutations in *plexA* and *Sema-1a*. The right schematic shows the effect of *Sema-2b* loss-of-function on the *sema2b*-positive neuron. The longitudinal axon is often seen moving from the middle to the lateral mAb 1D4-positive fascicle (shown on the right side of the embryo) and also to form an ectopic ventral projection, growing perpendicular to the plane of the longitudinal axons. anterior commissure (AC), posterior commissure (PC).

How do we understand these phenotypes with regard to a possible cell-autonomous function of *Sema-2b* in terms of Semaphorin-Plexin signaling? These data are consistent with the general model proposed by Moret et al. (2007), in which the cell-autonomous expression of secreted semaphorins may modulate the sensitivity of a growth cone to extracellularly presented semaphorins. While this could not occur in the same manner as in the chicken embryo due to the fact that *Drosophila* appears to lack a neuropilin receptor, it could act analogously on the Plexin receptors or a transmembrane semaphorin acting as a receptor. In the VNC, the misexpression phenotype of *Sema-2b* phenocopies that of *sema1a* and *plexA* loss-of-function mutants, but not *plexB* loss-of-function mutants (Fig. 6A). This suggests that there may be some specificity to the effects of *Sema-2b* for inhibition of *plexA* and *sema1a*. Given that *Sema-2b* can bind PlexB and not PlexA (Wu et al., 2011), it is possible that signaling downstream of PlexB inhibits the PlexA pathway. Previous work has found that the two Plexin proteins physically interact and this could underlie such an inhibitory mechanism (Ayoob et al., 2006). Furthermore, Sema-2a protein has been shown to be transported down axons suggesting that it could be available to control signaling in the growth cone (Sweeney et al., 2011). It has also been suggested that Sema-2b plays a role in promoting fasciculation (Wu et al., 2011). However, the ectopic ventral projection phenotype we observe in *Sema-2b* loss-of-function alleles does not appear to be simply aberrant defasciculation. The axons in these conspicuous and apparently fasciculated axon bundles leave the medial longitudinal fascicle into a region of the VNC that lacks pre-existing axon tracks and head ventrally, a direction that longitudinal axons never go. This suggests that Sema-2b is serving to actively and specifically restrict the directional options of longitudinal axons, and that the main effect may be directional and not simply an influence on axonal fasciculation.

The complexity of the adult nervous system is at least partially controlled by the action of axon guidance factors during development. To generate unique axon guidance behaviors among nearby neurons, differential gene expression of non-cell-autonomous and cell-autonomous factors is necessary. The identification of the *plexA, plexB*, and *sema1a* genes and their specific phenotypes suggests that these are important molecules in generating unique axon wiring in *Drosophila*. However, the apparently pan-neuronal expression of these factors does not immediately suggest an explanation for how these factors accomplish their function. The highly differential expression of *Sema-2b* allows another layer of regulation to produce axons that may have different sensitivities to semaphorin signaling.

## EXPERIMENTAL PROCEDURES

### Genetics

The 24B-Gal4, elav-Gal4 line was generated by standard recombination methods and verified for appropriate Gal4 expression by crossing to a β-galactosidase reporter line and assessing reporter expression by immunohistochemical detection of β-galactosidase. Df7142, f02042, and d05894 embryos were generated and obtained from Exelixis pharmaceuticals and were rebalanced over CyO,P[wg-lacZ] for immunohistochemistry purposes. Excision alleles of the f02042 element were generated using a source of piggyBac transposase obtained from the Bloomington Stock Center and applying standard techniques. The misexpression P-element EP2056 line was obtained from the Bloomington stock center and the ME722 line was generated in a P-element autosomal mobilization scheme using P[Mae-UAS.6.11] (Merriam et al., 1997). *Sema-2b* cDNA was generated from the RNA of embryos with the genotype w;1407-Gal4/ME722 and amplified with primers targeted to the start codon and stop codon of the predicted Sema-2b product. RT-PCR products were cloned into PGemTeasy (Promega, Madison, WI) and a product with the predicted Sema-2b full-length as well as three other products were sequenced fully and cloned into pUASt. The three other products were predicted to encode truncated Sema-2b protein products as a result of frameshifts caused by differential splicing.

### Immunohistochemistry

Embryo immunohistochemistry was performed according to Van Vactor and Kopczynski (1999). Embryos were incubated in mouse monoclonal 1D4 [Developmental Studies Hybridoma Bank (DSHB), Iowa City, IA] at a 1:5 dilution and rabbit anti-β-galactosidase (Cappel, ICN Pharmaceuticals, Aurora, OH) at a 1:5,000 dilution overnight at 4°C. Goat anti-mouse HRP and Goat anti-rabbit HRP antibodies (Jackson Immunoresearch, West Grove, PA) were used at 1:500 overnight at 4°C. To perform the misexpression screen, embryos from a cross of 50 to 60 virgin, female, homozygous 24B-Gal4, elav-Gal4, and 10 to 20 UAS misexpression P-element/CyO, P[UAS-LacZ] or TM3, Sb, P[UAS-LacZ] were fixed, incubated with the monoclonal 1D4, and immunohistochemically processed using a Vectastain DAB kit (Vector Labs, Burlingame, CA). Twelve embryos of each genotype were placed on a slide and oriented with either their VNC up or to the left. These whole-mounted embryos were cover-slipped and viewed under high magnification, allowing resolution close to that of dissected embryos. For immunofluorescence experiments, rabbit anti-Tau (1:500, Dako, Cambridgeshire, UK), 1D4 (1:50, DSHB), and monoclonal, anti-β-galactosidase 40-1a **(**1:50**)** were used with Goat anti-mouse 488 and Goat anti-rabbit 568 (Invitrogen, Carlsbad, CA). For phenotypic quantitation, embryos were dissected before being analyzed and embryos were scored blind with respect to genotype.

### Microscopy

Brightfield images of dissected embryos and phenotypic scoring were performed on an AxioPlanII microscope (Carl Zeiss Inc., Thornwood, NY). Brightfield images were captured with a Spot-RT camera (Diagnostic Instruments, Sterling Heights, MI) using Openlab3 software (Improvision, Waltham, MA). Confocal images were obtained with a Bio-Rad Radiance confocal microscope (Bio-Rad, Hercules, CA) with a Nikon E800 platform (Nikon, Tokyo, Japan). Image processing was performed with Velocity software (Improvision).

### RNA In Situ Hybridizations

RNA in situ hybridizations were carried out using a modified protocol (Rubin lab protocol book) from that developed by Tautz and Pfeifle (1989) that employs digoxigenin-labelled, single-stranded DNA probes. Most notably, the first 5 post-hybridization washes were done at 45°C.

### Quantitative RT-PCR (qRT-PCR) Analysis

To evaluate changes in *Sema-2b* RNA levels, total RNA was harvested from late stage embryos (stage 16–17). Approximately 200 embryos per sample were dechorionated in 50% bleach and RNA was isolated using Trizol reagent (Invitrogen, Life Technologies, Grand Isoland, NY). cDNA was reverse transcribed (iScript, Bio-Rad Laboratories, Hercules, CA) and qRT-PCR was carried out on a Life Technologies 7900HT (Life Technologies) machine using Power SYBR Green PCR Master Mix (Applied Biosystems, Life Technologies). Primers were designed using PrimerQuest (Integrated DNA Technologies, Coralville, IA; Forward *Sema-2b* primer: CCCAAAGCGAGTGTGTTTATTT, Reverse *Sema-2b* primer: GACGCATTCGTTTCCGTTTC). *Rps38* was used as an internal control to normalize samples (Forward *Rps38* primer: TTGCTATGGTGTGCTCCGCTACAT, Reverse *Rps38* primer: ACGAATTTCATCGACTTGGCACGC). qRT-PCR was performed for 37 cycles and, following amplification, melt curve analysis and agarose gel electrophoresis were performed to evaluate PCR products. Relative quantification of fold-change in mRNA expression was calculated using the 2-DDCT method.
